# A Rare Presentation of Multicanal Benign Paroxysmal Positional Vertigo in a Premenopausal Woman With Osteopenia: A Case Report

**DOI:** 10.7759/cureus.55421

**Published:** 2024-03-03

**Authors:** Maleeha S Abedi, Tania S Flink, Courtney P Roca

**Affiliations:** 1 Medicine, Lake Erie College of Osteopathic Medicine, Bradenton, USA; 2 Physiology, Lake Erie College of Osteopathic Medicine, Bradenton, USA; 3 Physical Therapy, Gannon University, Erie, USA

**Keywords:** canalith repositioning, premenopausal, canalithiasis, recurrence, multicanal, osteopenia, vitamin-d, benign paroxysmal positional vertigo

## Abstract

We report a case of non-traumatic, multicanal benign paroxysmal positional vertigo (BPPV) in a premenopausal, osteopenic 35-year-old female with corresponding low bone mineral density. Dix-Hallpike and supine roll tests confirmed unilateral posterior canal (PC) BPPV from 2012-2014, and later, a rare presentation of multicanal BPPV with specifically ipsilateral horizontal canals (HC) and anterior canals (AC) affected in 2015. Heel scans displayed T-scores within the osteopenia range in 2012 until levels normalized one year later. Despite treatment with indicated canalith repositioning treatments (CRTs), symptoms continued to persist. Complete resolution of symptoms occurred in 2016, which is most likely due to self-treatment with daily 5000 IU vitamin D in 2015. This case emphasizes the rare presentation of unilateral single-canal BPPV to multi-canal BPPV, along with the importance of vitamin D treatment in preventing the recurrence of symptoms.

## Introduction

Benign paroxysmal positional vertigo (BPPV) is the most common cause of vertigo, accounting for 10-27% of vertigo diagnoses [1]. With a lifetime prevalence of 2.4%, it is usually isolated and idiopathic in 80% of cases [2]. BPPV is believed to be caused by the dislodgement of otoconia into the semicircular canals, with the posterior canal (PC) most affected [3]. In rare cases, multiple canals may be affected, accounting for 4.7-12.2% of BPPV cases [4]. Compared to single canal BPPV, multicanal BPPV is more commonly associated with head trauma [4,5] 

There is an increased incidence of BPPV in women, corresponding with increased age and menopause [6]. There is a rapid decrease in estrogen receptors, which may disrupt otoconial metabolism. Estrogen can affect intestinal vitamin D absorption, which is disrupted when serum 25-OH is low [6]. Comorbidities such as hypertension, cardiovascular disease, and diabetes are also common in BPPV patients [7]. 

For this case report, a description and timeline on the presentation and treatment of BPPV in a 35-year-old female patient is included. The significance of this case report was the presentation of non-traumatic multicanal BPPV in a premenopausal, osteopenic woman with corresponding low bone mineral density (BMD) scans. Cessation of symptoms and potential treatment with vitamin D supplementation is discussed.

## Case presentation

The patient was a 35-year-old Caucasian female. The patient was healthy and at a normal weight, with a computed body mass index of 21.5. The patient reported no alcohol, tobacco, or drug use. Current medications included daily oral contraceptives (100 micrograms/20 micrograms levonorgestrel/ethinyl estradiol). No perimenopausal symptoms or menstrual irregularities were reported at the time. The patient reported occasional migraine headaches but had not been diagnosed with hypertension, diabetes, or hyperlipidemia. She had no history of head trauma. The patient was an avid runner, running seven half-marathons between 2013-2018. The patient trained for a race in the spring of 2014 but was not able to compete due to recurrent BPPV symptoms. 

**Figure 1:**
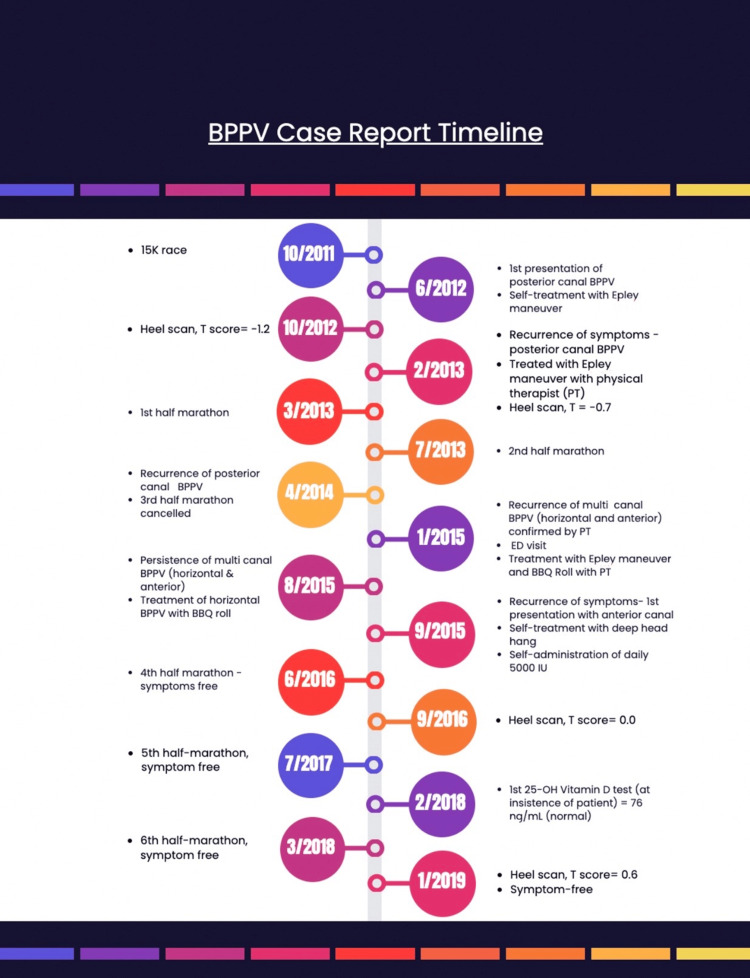
Timeline of progression of BPPV symptoms, diagnosis, and treatments, along with pertinent health information and tests.

The patient first presented with unilateral BPPV in the PC on the left side in June 2012. A timeline of events is presented in Figure 1. Symptoms included brief, episodic, positional vertigo with vertical head tilt, clamminess, nausea, and headache. The patient self-diagnosed herself with PC canalithiasis BPPV and successfully self-performed an Epley maneuver twice to assuage symptoms. The patient remained symptom-free until March 2013; at this time, the PC canalithiasis BPPV symptoms recurred on the left side with greater intensity. The patient sought help from a physical therapist (PT), who confirmed the PC canalithiasis diagnosis with the left Dix-Hallpike test, demonstrating latent and brief up-beating left torsional nystagmus. The PT successfully treated the PC canalithiasis BPPV using the Epley maneuver in two sessions of three maneuvers each. A second recurrence of PC BPPV on the left side occurred in April 2014. The patient, at this time, was referred to a PT specializing in vestibular disorders. PC canalithiasis BPPV on the left side was confirmed again with the Dix-Hallpike test using videonystagmography, as demonstrated by latent and brief up-beating left torsional nystagmus. The patient was treated successfully with the Epley maneuver with the vestibular PT in two sessions of three maneuvers each.

In January 2015, the first presentation of multicanal BPPV occurred. The patient was initially admitted to the emergency department with debilitating vertigo, which was of greater intensity than previously experienced. Complete blood count, metabolic panel numbers, and review of systems were normal. The patient did not have a fever, and a non-contrast computed tomography (CT) scan of the head was negative. A diagnosis of positional vertigo was given at discharge (no specific canal specified). The patient was also prescribed ondansetron (4 mg) and meclizine (25 mg) PRN with orders to continue care with the previous vestibular PT on an outpatient basis. Ondansetron and meclizine were not taken prior to the rehabilitation sessions. The subsequent PT sessions revealed a presentation of multicanal canalithiasis BPPV with horizontal canal (HC) and anterior canal (AC) involvement on the left side. Videonystagmography revealed HC involvement as evidenced by geotropic nystagmus bilaterally (left > right) during the supine roll test. The Dix-Hallpike test confirmed left-side AC involvement as evidenced by latent and brief down-beating left torsional nystagmus. The HC was treated first with the barbecue roll maneuver, followed by the deep head hanging maneuver for AC canalithiasis BPPV. The symptoms lessened after six sessions, specifically for the HC canalithiasis BPPV (Figure 1).

The patient was referred to an Ear, Nose, and Throat (ENT) specialist via her primary care physician in May 2015. At this time, the patient continued to experience symptoms associated with BPPV with horizontal and anterior movements, but symptoms were mild. Auditory brainstem evoked responses, electrocochleography, and audiology tests were normal, and the Dix-Hallpike test was negative. Electronystagmography during caloric testing revealed a 32% directional preponderance to the left and an indication of peripheral and central vestibular abnormalities. Symptoms of HC canalithiasis BPPV worsened on the left side in August 2015, and the patient commenced self-administration of the barbecue roll. This was successfully treated with 2-3 daily maneuvers for 5 days. AC BPPV symptoms continued to persist until September-October 2015, which included vertigo with forward head movements in the sagittal plane. Self-treatment with the Epley maneuver for this canal was not successful. As such, the patient self-initiated treatment with the deep head hang maneuver. The AC BPPV was treated successfully after 2-3 days, and the BPPV symptoms did not recur. 

**Figure 2:**
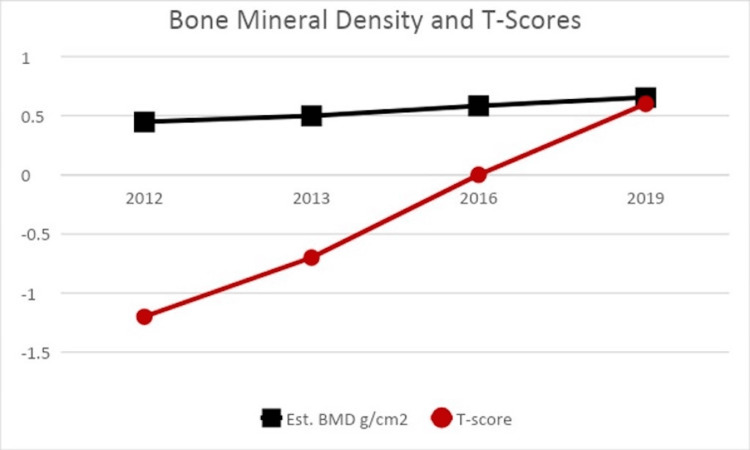
Estimated bone mineral density (BMD) and T-scores measured from 2012-2019 using heel sonography.

In September 2015, the patient also began consuming 5000 IU vitamin D3 supplements daily. This was at the suggestion of the vestibular PT, in addition to research self-initiated by the patient. The patient was symptom-free as of 2016. In February 2018, at the patient's request, 25-OH vitamin D levels were measured for the first time and were found to be 76 ng/mL (normal reference range = 30-100). Due to its availability and personal interest, the patient also collected bone density data using a heel sonometer from 2012 to 2019. BMD and T-scores were recorded on her right heel. The data are summarized in Figure 2. In 2012, the patient's T-scores were within the osteopenia range but increased to within normal limits by 2019. BMD increased by 46% from 2012-2019.

## Discussion

BPPV is the most common peripheral vestibular disease that causes recurrent episodes of vertigo and dizziness that worsen with head movement [8,9]. Calcium carbonate crystals, or otoconia, are continuously remodeled, requiring calcium and regulation by vitamin D [10]. When this is disrupted, it may cause dislodgement of the otoconia from the otolith organs and deposition in the semicircular canals [1,10,11]. Associated nystagmus depends on the semicircular canals involved, which can be elicited by specific positional tests such as the Dix-Hallpike and supine roll test [12]. Unilateral PC canalithiasis is the most common presentation of BPPV [9,12,13]. This is due to the anatomical location of the PC semicircular canal at the most dependent position in a standing person, which frequently causes the deposition of otoconia into the canal [13]. In rare cases, multiple semicircular canals, known as multicanal BPPV, may be involved, which has a lower prevalence compared to single canal BPPV [4,8].

Compared to single canal, multicanal BPPV is more likely associated with head trauma, found in up to 30% of cases [5,8,14]. Additionally, patients with multicanal BPPV are typically older compared to patients with single canal BPPV. In addition, those with multicanal BPPV are more likely to have an underlying vestibular condition, such as labyrinthitis [8,14]. However, for this case study, head trauma and other underlying vestibular conditions (labyrinthitis, Meniere's disease, vestibular neuritis) were not present in the patient as evidenced by the non-contrast CT scan performed during the emergency department visit and the vestibular data from the ENT visit.

The most common presentation of multicanal BPPV is ipsilateral PC and HC canalithiasis BPPV [3,8]. Mixed torsional geotropic-vertical upbeating nystagmus in Dix-Hallpike maneuvers reveals involvement of PC, whereas, geotropic or apogeotropic horizontal nystagmus during supine roll test reveals involvement of HC [3]. The combination of HC and AC canalithiasis BPPV is incredibly rare due to the involvement of the AC, which occurs in 2-3% of all cases [4]. For this case study, the patient was first presented with unilateral single BPPV in the PC of the left side but progressed to a multicanal presentation (HC and AC). This presentation is rare, and it is likely attributed to canal conversion during canalith repositioning treatments in non-traumatic patients [14].

Regardless of the number of canals involved, BPPV has increased incidence and recurrence in aged populations and females [15]. Advancing age leads to progressive demineralization of the otoconia, which becomes pitted and fissured, causing eventual degradation, fragmentation, and detachment from the otolith organs [13,16,17]. The recurrence of BPPV is increased in patients older than 65 years of age and is much higher; it may be due to the reduction in daily living, mobility, and balance activities [13,17]. In this case, the patient is considerably younger (35 years) compared to the average age of presentation, which ranged from 48 to 62 years [6]. In addition, the patient was not sedentary and trained and participated in several half-marathons. Therefore, age and activity levels are not likely to be contributing risk factors for BPPV in this case.

The presence of comorbidities such as hypertension, vascular disease, and diabetes may also increase the rate of recurrence of BPPV, especially in aged patients [13,17]. The vestibular system degrades with age, and reduced blood flow caused by hypertension and vascular disorders can cause otoconial detachment and subsequent deposition [17]. In addition, higher recurrence of BPPV is noted in individuals with a history of diabetes, which causes increased vascular resistance, neuropathy, and vasculopathy, leading to cupular deposits and floating debris commonly in PCs and HCs [13,17]. Hyperinsulinism may disrupt inner ear homeostasis because of the large number of insulin receptors present in the endolymphatic sac [17]. For this patient, the complete blood count, metabolic panel, and general health history indicated no presence of comorbidities such as hypertension, vascular disease, and diabetes during the multicanal presentation at the emergency department. Therefore, these comorbidities are not likely to be contributing factors for the multicanal BPPV presentation in this case.

In women, hormonal changes, external estrogens, and osteopenia/osteoporosis can be possible contributors to the predominance of BPPV compared to men. Estrogen declines dramatically during menopause; it plays a significant role in the inner ear and otoconial metabolism. Estrogen also plays a role in the development of osteopenia and osteoporosis. Loss of reproductive function and aging are the two significant factors. After ages 30-40, men and women experience 0.3-0.5% bone loss; however, after menopause, the rate of bone loss can increase 10-fold [6]. It is characterized by low BMD and deterioration of bone tissue, resulting in bone fragility [18]. Estrogen affects bone through calcium metabolism; specifically, it increases the sensitivity of bone mass to parathyroid hormone, decreasing calcitonin production and intestinal calcium resorption and increasing calcium excretion, which ultimately causes less binding to estrogen receptors in the bone [16,18]. Using bone scanning methodology, osteopenia is defined as a measured T-score between -1.0 and -2.5 [18]. For this case report, the patient initially presented with osteopenia at the time of first symptom onset, with a T-score measurement of -1.2 using a bone sonometer of the right heel. At the time of the first symptom onset, the patient had been taking daily oral contraceptives (100 micrograms/20 micrograms levonorgestrel/ethinyl estradiol) for over 16 years. In addition, the patient had been actively training for half marathon races. While the exact causes for the osteopenia reading are unknown, they are not likely due to advanced age, reduced estrogen level, or lack of physical activity. It was therefore suggested that the low BMD may be due to other external factors, such as dietary deficiency, which is described next.

Otoconia consist of calcium carbonate and are continuously remodeled throughout the lifetime [3,13]. Calcium and carbonate levels in the endolymph are regulated to maintain mineralization, which occurs due to an opening on the crystalline surface of otoconia. Vitamin D plays a crucial role in the body's calcium homeostasis, specifically in the inner ear, by keeping the calcium concentration at a critical level for normal otoconial development. Vitamin D levels are considered normal with serum levels of 25(OH)D at 25-80 ng/mL. Insufficiency is defined as serum levels of 25-OH(D) less than 30 ng/mL, deficiency as less than 20 ng/mL, and severity as less than 10 ng/mL [2,16]. In the presence of deficiency, treatment with vitamin D normally begins.

Vitamin D deficiency may also contribute to the occurrence and recurrence of BPPV by affecting calcium metabolism [17]. Deficiency may cause down-regulation of calcium-binding proteins and epithelial transport systems, directly affecting otoconial production and repair [17]. It has been proposed that vitamin D deficiency can lead to increased incidence of BPPV, whereas correction of vitamin D levels may reduce BPPV recurrence, with doses of vitamin D ranging from 10,000 to 50,000 IU weekly or biweekly and gradually decreasing the dose over several months [1,2,6].

The patient in question began taking 5000 IU of vitamin D3 in the fall of 2015 at the suggestion of her vestibular PT and after personal research on the issue. There were no recurrences of symptoms hereafter. 25-OH vitamin D levels were within the normal range in February 2018, and a rescan of the heel with the sonometer yielded a T-score in a healthy range in January 2019. This suggests that an initial vitamin D deficiency was the most likely risk factor that could have contributed to the initial presentation of PC canalithiasis BPPV symptoms and the subsequent multicanal recurrences that occurred for several years afterward. An initial measurement of 25-OH vitamin D levels first presented in 2012 would confirm this hypothesis, but practitioners did not prescribe testing at that time.

## Conclusions

The current study highlights the rare presentation of multicanal BPPV in a younger patient with osteopenia and adds to the literature information on the manifestations, risk factors, and management of multicanal BPPV. For this case report, age, comorbidities, and low estrogen were insignificant risk factors that led to the presentation of BPPV. Treatment of unilateral canalithiasis using standard canalith repositioning treatments may have led to multicanal presentation as a result of canal conversion. However, deficiency in vitamin D may have also played a role in the initial presentation of the disorder and the subsequent recurrences, particularly in the absence of other comorbidities or other risk factors. 

This case highlights the significant impact of vitamin D deficiency on the occurrence and recurrence of BPPV and the rare presentation of multicanal BPPV. The recommended dose of vitamin D is 800 to 1000 IU daily, but in patients with severe deficiency, the prescribed amount may be higher. Therefore, yearly screening for serum 25-OH to determine vitamin D levels in the general population is suggested, as early and continuous administration of vitamin D may be valuable approaches for preventing idiopathic BPPV in the general population.
